# Relationships between Head Circumference, Brain Volume and Cognition in Children with Prenatal Alcohol Exposure

**DOI:** 10.1371/journal.pone.0150370

**Published:** 2016-02-29

**Authors:** Sarah Treit, Dongming Zhou, Albert E. Chudley, Gail Andrew, Carmen Rasmussen, Sarah M. Nikkel, Dawa Samdup, Ana Hanlon-Dearman, Christine Loock, Christian Beaulieu

**Affiliations:** 1 Neuroscience and Mental Health Institute, University of Alberta, Edmonton, Alberta, Canada; 2 Department of Biomedical Engineering, University of Alberta, Edmonton, Alberta, Canada; 3 Departments of Pediatrics and Child Health and Biochemistry and Medical Genetics, University of Manitoba, Winnipeg, Manitoba, Canada; 4 Department of Pediatrics, University of Alberta, Edmonton, Alberta, Canada; 5 FASD Diagnostic Clinic, Glenrose Rehabilitation Hospital, Edmonton, Alberta, Canada; 6 Department of Pediatrics, University of Ottawa, Ottawa, Ontario, Canada; 7 Department of Pediatrics, Queens University, Kingston, Ontario, Canada; 8 Department of Pediatrics and Child Health, University of Manitoba, Winnipeg, Manitoba, Canada; 9 Department of Pediatrics, University of British Columbia and Sunny Hill Health Centre for Children, Vancouver, British Columbia, Canada; Indiana University, UNITED STATES

## Abstract

Head circumference is used together with other measures as a proxy for central nervous system damage in the diagnosis of fetal alcohol spectrum disorders, yet the relationship between head circumference and brain volume has not been investigated in this population. The objective of this study is to characterize the relationship between head circumference, brain volume and cognitive performance in a large sample of children with prenatal alcohol exposure (n = 144) and healthy controls (n = 145), aged 5–19 years. All participants underwent magnetic resonance imaging to yield brain volumes and head circumference, normalized to control for age and sex. Mean head circumference, brain volume, and cognitive scores were significantly reduced in the prenatal alcohol exposure group relative to controls, albeit with considerable overlap between groups. Males with prenatal alcohol exposure had reductions in all three measures, whereas females with prenatal alcohol exposure had reduced brain volumes and cognitive scores, but no difference in head circumference relative to controls. Microcephaly (defined here as head circumference ≤ 3rd percentile) occurred more often in prenatal alcohol exposed participants than controls, but 90% of the exposed sample had head circumferences above this clinical cutoff indicating that head circumference is not a sensitive marker of prenatal alcohol exposure. Normalized head circumference and brain volume were positively correlated in both groups, and subjects with very low head circumference typically had below-average brain volumes. Conversely, over half of the subjects with very low brain volumes had normal head circumferences, which may stem from differential effects of alcohol on the skeletal and nervous systems. There were no significant correlations between head circumference and any cognitive score. These findings confirm group-level reductions in head circumference and increased rates of microcephaly in children with prenatal alcohol exposure, but raise concerns about the predictive value of this metric at an individual-subject level.

## Introduction

Head circumference (HC) trajectories are commonly used as a gross measure of neurological development in infancy and early childhood, providing a rapid and cost-effective means to screen for abnormalities such as hydrocephalus or delayed development [1]. Although not typically used in routine care beyond 3 years of age, HC is shown to positively correlate with brain volume in groups of healthy children and adolescents. However, this relationship is noted to weaken after the age of 7 years, likely given that total brain volume expansion peaks early in childhood while skull thickness and non-neural tissue growth continue throughout adolescence [2,3]. HC is often smaller in children with mental retardation [4] and has been correlated with intelligence in healthy populations [3], though relationships between HC and cognitive ability appear to be less consistent [5–7]. Nonetheless, large deviations from the norm at any age can indicate micro- or macrocephaly associated with a multitude of genetic disorders, perinatal brain injuries, and teratogenic exposures [8,9] including prenatal alcohol exposure (PAE).

Neurotoxicity from prenatal alcohol exposure is sometimes observable at birth, as evidenced by reduced birth weight [10], lower Apgar scores [11] and increased rates of microcephaly (low HC) in infants with PAE [12]. HC reductions persist throughout childhood and adolescence, and one study has shown correlations between HC and performance IQ in 8–16 year olds with PAE [13]. Several studies of PAE suggest that the degree of HC reduction relates to timing [12], amount [14,15] and pattern [16,17] of alcohol exposure in utero, though HC reductions are typically modest (e.g. -1.3 to -3.9% in children of heavy drinkers compared to abstainers [14,16,18]) and are not present in every sample [19].

Nonetheless, HC is used (among other measures) in the diagnosis of fetal alcohol spectrum disorders (FASD) as “evidence of deficient brain growth or abnormal morphogenesis”[20], “structural evidence of CNS damage”[21] or “structural CNS dysfunction”[22]. However, the relationship between HC and brain volume has not been reported in children with PAE and may differ from observations in typically developing children or children with microcephaly of other etiologies. Further investigation of this relationship is needed to better characterize the clinical significance of microcephaly in children with PAE and to inform the use of HC in FASD diagnostic guidelines.

## Methods

### Participants

This study was approved by the Health Research Ethics Boards at the University of British Columbia, University of Alberta, University of Manitoba and Queens University. Participants were 144 individuals with confirmed PAE (5–19 years, mean 12.5 ± 3.3 years; 76 males) and 145 controls (5–19 years, mean 11.9 ± 3.4 years; 69 males). This subject pool includes participants from previous [23–25] and current FASD studies conducted at the University of Alberta, as well as from a new multi-site MRI study of brain development [26]. PAE participants were recruited through various multi-disciplinary FASD diagnostic clinics across Canada, had confirmed prenatal alcohol exposure and were assessed according to the Canadian Guidelines for the Diagnosis of FASD [22] and the 4-Digit Code [21]. Of the 144 participants in the PAE group, 33 (23%) had a dysmorphic diagnosis of fetal alcohol syndrome (FAS) or partial fetal alcohol syndrome (pFAS), 79 (55%) were diagnosed with static encephalopathy: alcohol exposed (SE:AE), neurobehavioural disorder: alcohol exposed (NBD:AE), alcohol related neurodevelopmental disorder (ARND), or FASD that was not further specified, and 32 (22%) had confirmed pre-natal alcohol exposure but did not meet criteria for formal diagnosis or were deferred for re-evaluation. Controls were recruited through advertising and had no self-reported history of neurological, psychiatric, or developmental disorders. All participants or parents/legal guardians provided written informed consent, and participants were screened for contraindications to MRI.

### Image Acquisition and Analysis

Head circumference and brain volumes were calculated from magnetic resonance imaging (MRI) scans (T1-weighted 3D-MPRAGE, 1x1x1 mm^3^) collected on 4 scanners across Canada: 3T Philips Intera at University of British Columbia (12 PAE and 16 controls), 1.5T Siemens Sonata at University of Alberta (100 PAE and 106 controls), 3T Siemens Trio at each of University of Manitoba (9 PAE and 8 controls) and Queens University (23 PAE and 15 controls). Total brain volume (excluding brainstem, cerebellum and cerebrospinal fluid) and the volume of the frontal, temporal, parietal and occipital lobes were calculated with Freesurfer v5.1, averaging left and right hemispheres. HC was manually traced by the same user (ST) on an axial oblique slice aligned with the most prominent parts of the occiput and forehead in OsiriX v5.8.5.

### Inter-site Reliability

Given that data were collected on multiple MRI scanners, reliability of HC and brain volume measurements between scanners was assessed with a ‘travelling phantom’ study. In short, 8 adult subjects were flown to all four sites to be scanned twice each (8 scans per person, 64 scans total, mean of 102 days from first to last scan), using the identical scanning protocol as this study. Imaging data was analyzed with identical methods as described here, and reliability was evaluated by computing the within and between-subject coefficient of variation (CV) and Intraclass Correlation Coefficients (ICC; absolute agreement) for brain volume and head circumference.

### Cognitive Testing

Cognitive testing was performed by a trained research assistant on the same day as each subjects’ MRI scan. The test battery included: Woodcock Johnson (WJ) Quantitative Concepts; Woodcock Reading Mastery Test-Revised (WRMT-R) Word ID; Working Memory Test Battery-Children (WMTB-C) Digit and Block recall; NEPSY-II Animal Sorting, Auditory Attention, Inhibition, and Memory for Names; Behavior Rating Inventory of Executive Function (BRIEF) Parent form and the Wide Range Intelligence Test (WRIT) General IQ (**Table 1**). For a small subset of PAE subjects (n = 12), Wechsler Intelligence Scale for Children (WISC) full scale IQ was instead collected via chart review.

**Table 1:** Subject Characteristics and Cognitive Test Scores.

		Control	PAE	PAE with HC ≤ 3^rd^ percentile^a^
Sample size		145	144	14
Age (years)		11.9 ± 3.4	12.5 ± 3.3	13.9 ± 3.3
Number of Males		69 (48%)	76 (53%)	8 (57%)
Ethnicity				
	Caucasian	126 (87%)	38 (26%)	4 (33%)
	Aboriginal	4 (3%)	72 (50%)	7 (50%)
	Other/Unknown	15 (10%)	34 (24%)	3 (25%)
Wide Range Intelligence Test/Weschler Intelligence Scale^c^				
	General IQ	112 ± 12 (n = 66)	88 ± 17** (n = 50)	—^e^
Woodcock Johnson^b^				
	Quantitative Concepts 18A&B	106 ± 15 (n = 141)	82 ± 19** (n = 122)	89 ± 18 (n = 11)
Woodcock Reading Mastery Test^b^				
	Word ID	106 ± 13 (n = 140)	90 ± 15** (n = 110)	93 ± 13 (n = 10)
BRIEF (parent form)^c^				
	Behavioural Regulation Index	48 ± 8 (n = 131)	72 ± 12** (n = 116)	76 ± 13 (n = 11)
	Metacognitive Index	51 ± 12 (n = 130)	68 ± 10** (n = 116)	70 ± 12 (n = 11)
	Global Executive Composite	49 ± 10 (n = 130)	73 ± 10** (n = 116)	75 ± 11 (n = 11)
Working Memory Test Battery^b^				
	Digit	99 ± 16 (n = 125)	85 ± 13** (n = 108)	88 ± 9 (n = 7)
	Block	100 ± 16 (n = 124)	86 ± 16** (106)	88 ± 15 (n = 8)
NEPSY-II^d^				
	Animal Sorting	9.4 ± 3.8 (n = 129)	7.0 ± 3.4** (n = 86)	8.3 ± 5.6 (n = 7)
	Auditory Attention	10.3 ± 3.1 (n = 135)	7.1 ± 4.1** (n = 103)	7.9 ± 5.1 (n = 9)
	Response Set	10.1 ± 3.5 (n = 129)	8.9 ± 3.9 (n = 101)	10.7 ± 2.8 (n = 9)
	Inhibition-Naming	9.3 ± 3.5 (n = 135)	6.6 ± 4.0** (n = 99)	8.4 ± 5.0 (n = 8)
	Inhibition-Inhibition	9.7 ± 3.8 (n = 135)	6.1 ± 3.6** (n = 98)	6.4 ± 4.2 (n = 8)
	Inhibition-Switching	10.2 ± 3.8 (n = 129)	6.4 ± 4.1** (n = 96)	6.4 ± 6.5 (n = 8)
	Memory for Names	9.3 ± 2.9 (n = 135)	6.1 ± 3.6** (n = 105)	6.7 ± 4.5 (n = 9)

**p<0.001 on independent sample t-tests (PAE versus Controls)

^a^Relative to population norms reported in Rollins et al Journal of Pediatrics, 2010 [1]. Significance versus controls not tested for HC≤3^rd^ percentile due to small sample size (n = 3)

^b^Standard scores, mean = 100, SD = 15, higher score indicates better performance

^c^T scores, mean = 50, SD = 10, higher score indicates worse performance

^d^Scaled scores, mean = 10, SD = 3, higher score indicates better performance

^e^Mean ± SD not reported given n = 3 with IQ scores in this category

### Normalization of Cognitive Scores, HC and Brain Volume

Raw cognitive scores were converted to standard, scaled or t-scores, according to the procedures outlined by each test manual. Raw HC values were converted to standard deviations (SDs) and percentiles based on a large population based sample [1], in order to control for age and sex. Given that there are no normative standards for brain volume, raw brain volumes were converted to Z scores based on the control group mean and standard deviation, calculated separately in males and females.

### Group Differences, Change with Age, and Correlations Between HC, Brain Volume and Cognition

Group differences (PAE vs control) in normed cognitive scores, raw HC and raw brain volume were determined with independent sample t-tests, while change with age was tested with Pearson’s correlations (alpha set to p<0.05); in these cases males and females were analyzed separately. Relationships between normalized HC (SD), brain volume Z scores, and cognitive test scores were explored with Pearson’s correlations, assessed separately for PAE and controls; note that the standard deviation and Z values have already factored sex into account.

In addition, correlations between normed HC and brain volume Z scores were then repeated including only subjects with HC at least 1 SD below the population norm (below the ~15^th^ percentile) to further test this relationship outside of the normal range. Although a diagnosis of microcephaly is not given unless HC is ≤ 10^th^ percentile [20] or ≤3^rd^ percentile [21,22], this more liberal cutoff was chosen to increase power in a subset of PAE participants on the low end of the HC spectrum (n = 34), given that the clinical cutoffs of the 10^th^ or 3^rd^ percentile would limit the sample to 22 and 14 participants, respectively. Instead descriptive statistics are presented for participants below these clinical cut-offs. Specifically, the proportion of subjects with HC ≤3^rd^ percentile, ≤10^th^ percentile, and 11^th^-99^th^ percentile that have brain volumes that are ≤3^rd^ percentile, ≤10^th^ percentile, and 11^th^-99^th^ percentile are reported separately, as well as proportions of subjects with brain volume HC ≤3^rd^ percentile, ≤10^th^ percentile, and 11^th^-99^th^ percentile who have head circumferences that are ≤3^rd^ percentile, ≤10^th^ percentile, and 11^th^-99^th^ percentile.

## Results

### Inter-site Reliability

**Fig 1:**
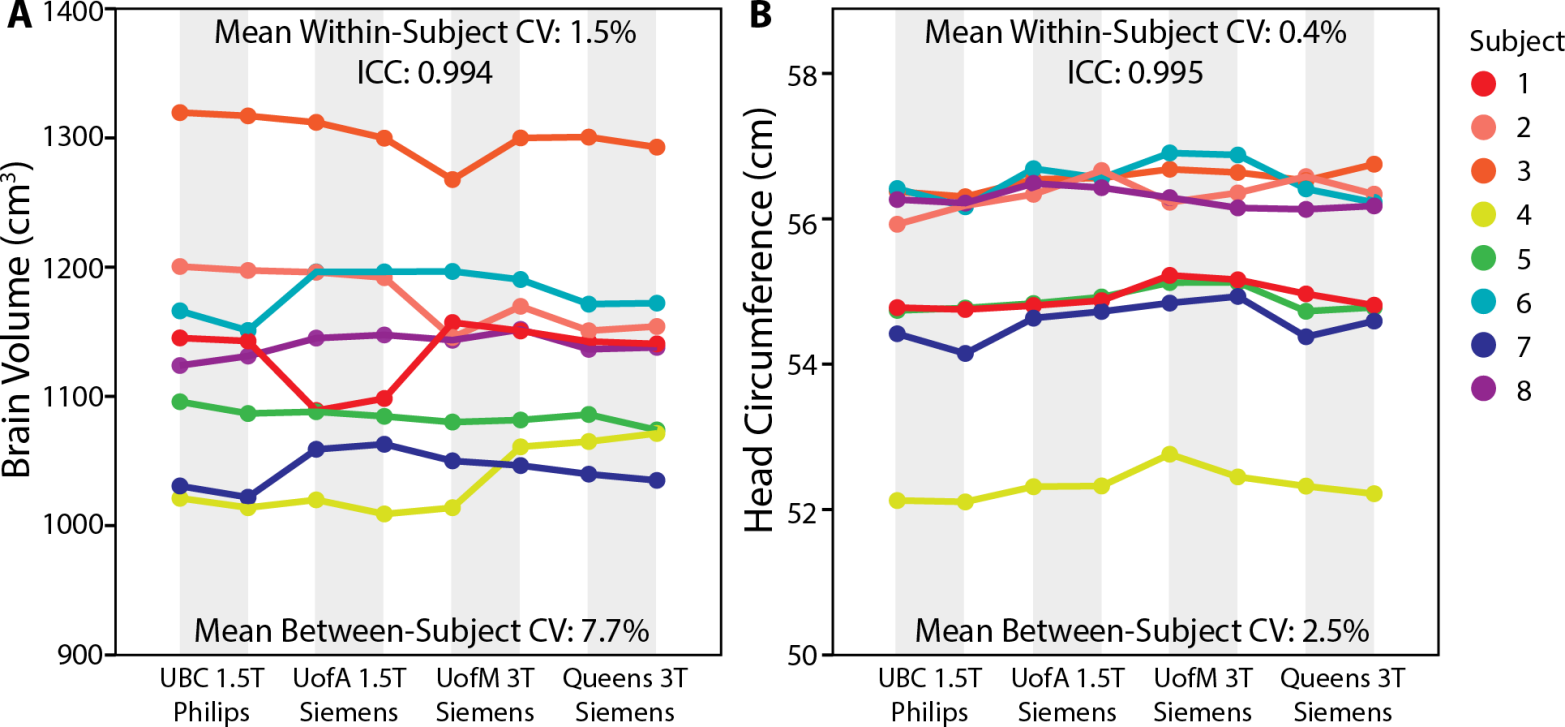
Inter-site reliability of (A) brain volume and (B) head circumference measurements from 8 adult subjects, scanned twice each at all four sites. For both brain volume and head circumference measurements, within-subject variability was much lower than between subject variability (as reflected by ICCs of 0.994 and 0.995, respectively) suggesting that data can be combined across scanners for these measures without obvious bias.

The travelling phantom study (of 8 adult subjects scanned twice each at each of the 4 sites) indicated excellent absolute agreement between scanners for both brain volume and head circumference, as reflected by ICCs of 0.994 and 0.995, respectively (**Fig 1**). Mean within-subject variability (across scanners) was 1.5% for brain volume and 0.4% for head circumference; for both measures this was roughly 5 times lower than the between-subject variability of 7.7% and 2.5%, respectively. These results indicate little effect of scanner on these measurements, and suggest that data can be combined across sites with limited risk of introducing site-related bias.

### Group Differences and Changes with Age

**Fig 2:**
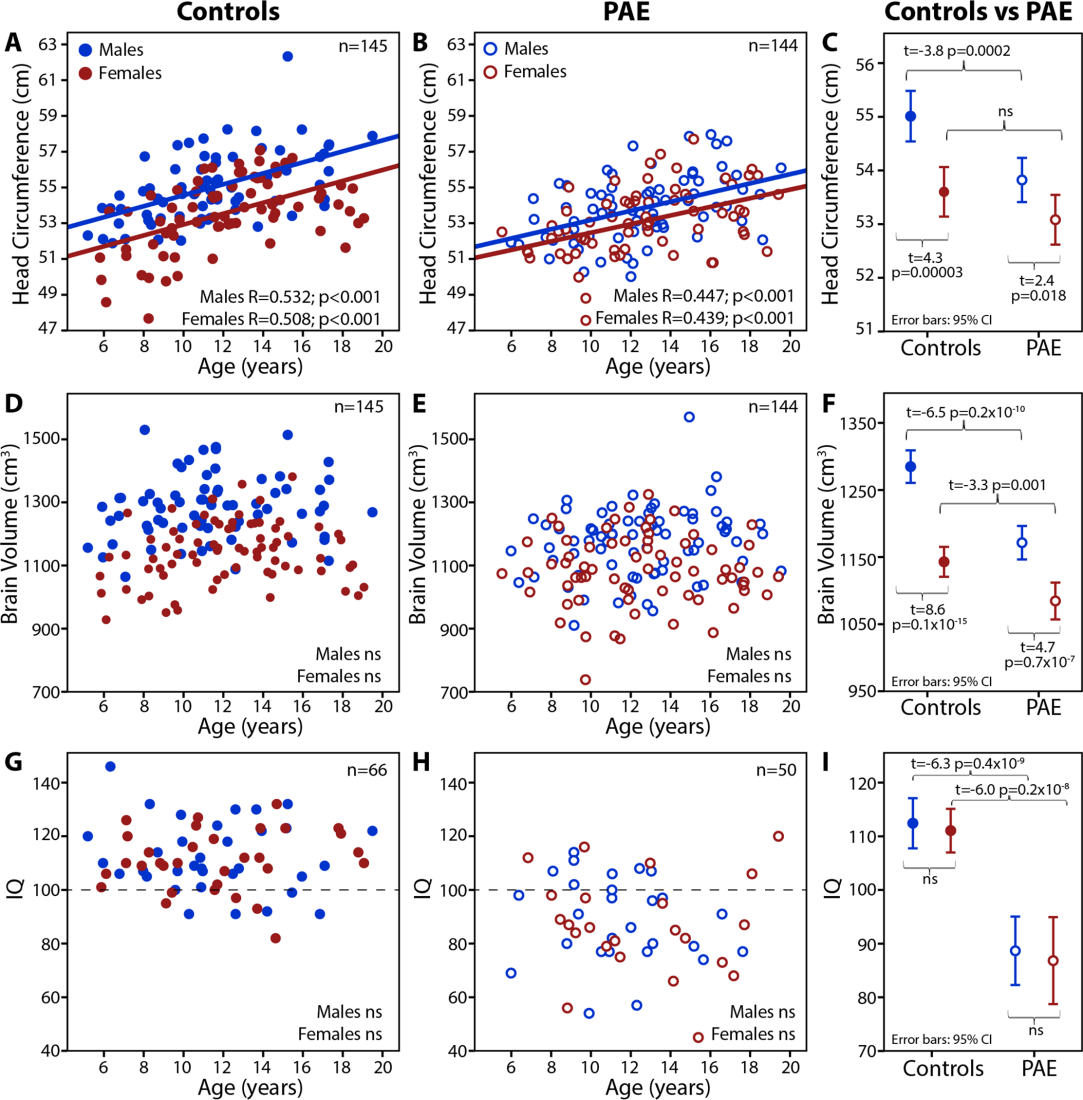
Head circumference increased with age for both males (blue) and females (red) in both groups (A, B), and HC was lower in PAE than controls for males but not females (C). Brain volume did not change with age in either group (D,E), and was consistently reduced in PAE relative to controls for both males and females (F). IQ standard scores did not change with age in either group (G, H), and were again lower in the PAE group (I). Sex differences within groups were larger in the control group for both head circumference (C) and brain volume (F). ns = non-significant.

Raw HC increased with age in both groups and sexes (male controls R = 0.53 p<0.001; female controls R = 0.51, p<0.001; male PAE R = 0.45, p<0.001; female PAE R = 0.44, p<0.001- **Fig 2A and 2B**), but raw brain volume did not change with age (**Fig 2D and 2E**). Raw HC was 2.2% lower in males with PAE versus male controls, but females with PAE did not differ from female controls (t = -3.81, p<0.001 and t = -1.58, p = 0.117, respectively—**Fig 2C**). Conversely, raw brain volume was lower in both males (-8.8%) and females (-5.1%) with PAE relative to controls (t = -6.47, p<0.001 and t = -3.32, p = 0.001, respectively—**Fig 2F**), though greater overlap between sexes can be seen in the PAE than control group (**Fig 2D and 2E**). In the subset of participants with IQ scores, IQ did not change with age (as expected for a standard score) and sex differences were not significant in either group (**Fig 2G–2I**), though the control group (IQ~112) significantly outperformed the PAE group (IQ~88) (t = -8.73, p<0.001- **Table 1**, **Fig 2I**). Likewise, the control group scored better than the PAE group on all other cognitive tests (t = -2.37–19.19, p<0.001–0.019—**Table 1**).

**Fig 3:**
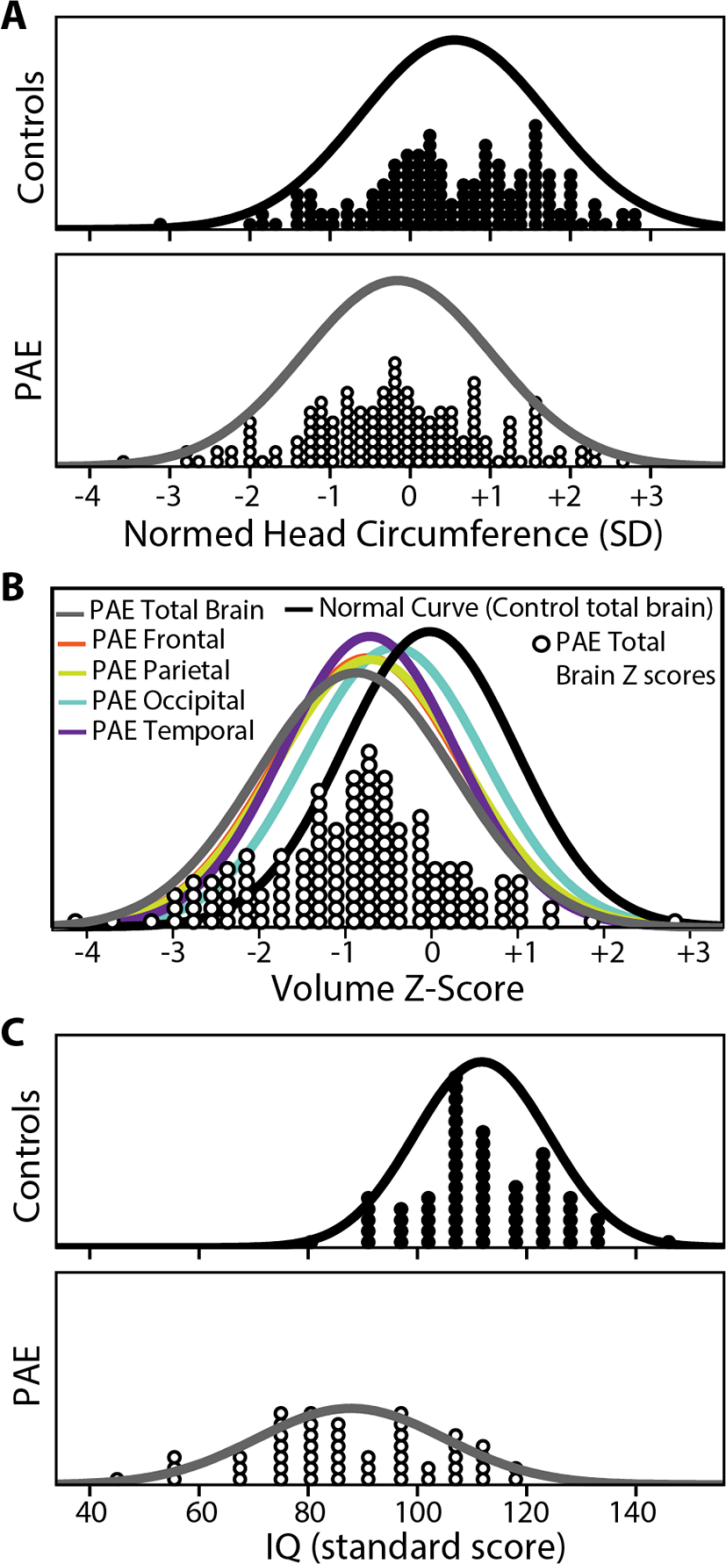
(A) Head circumference (HC) standard deviation distributions showing a shift towards the number of participants with higher normed HC in controls and lower normed HC in PAE subjects, albeit with substantial overlap between groups. (B) Z score distribution for total brain volume is left- shifted in the PAE group (grey curve) relative to controls (black curve). PAE distribution curves for brain lobe volumes show similar leftward shifts towards negative Z scores. (C) Likewise, IQ score profile is right-shifted in the control group compared to PAE (C), peaking above the population norm of 100 in controls, and below in the PAE group.

Normed HC, brain volume and IQ scores had right-shifted distributions toward higher values in controls relative to the lower values in the PAE group (**Fig 3A–3C**). Notably, only 10% of controls had a HC more than 1 SD below the population norm, compared to 24% of the PAE group. The greatest reductions of HC, IQ and brain volume were found in participants with dysmorphic features indicative of FAS/pFAS (data not shown) in keeping with previous literature [27]; however, subgroup analysis was not further explored given that IQ and HC are used in the sub-classification of FASD. Despite group differences, substantial overlap between the PAE and control groups is evident for all three metrics (**Figs 2 and 3**).

### Head Circumference Correlations

**Fig 4:**
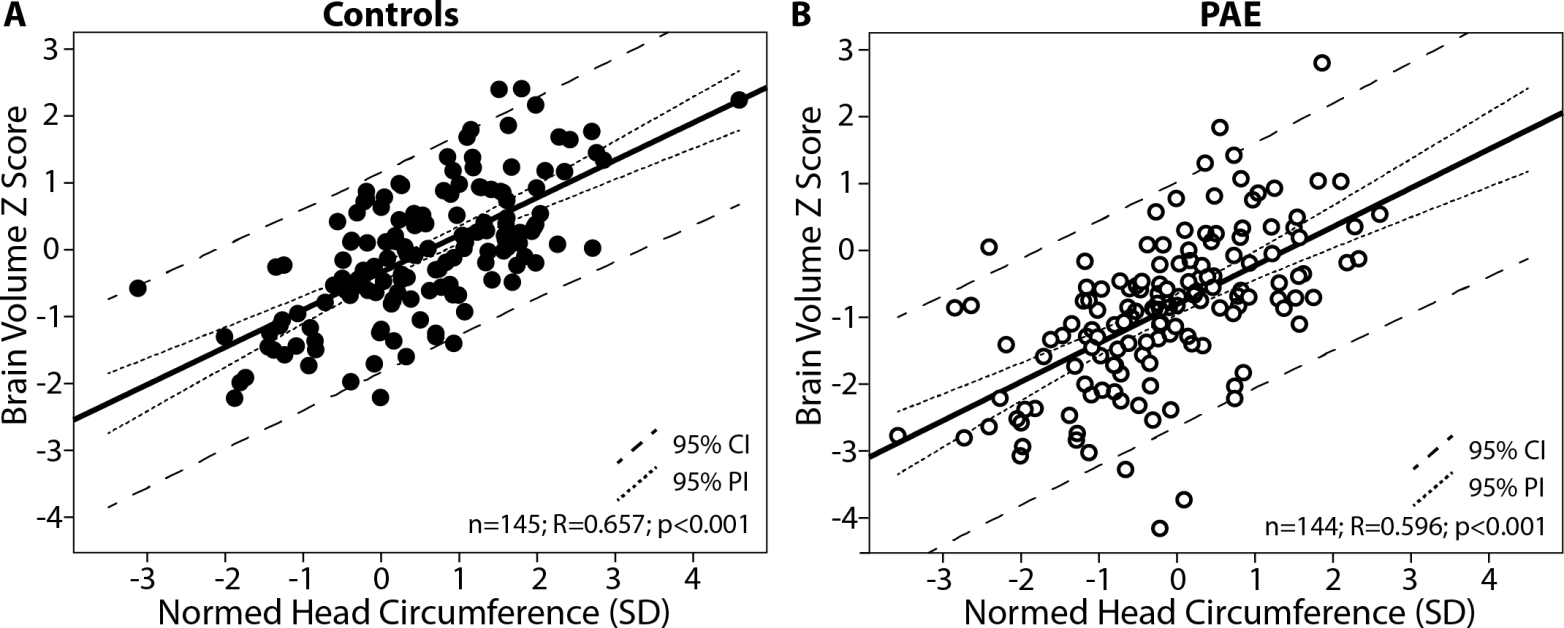
Brain volume Z scores and normed head circumference (HC) standard deviations are shown to positively correlate in both the control (A) and prenatal alcohol exposure (PAE) groups (B).

**Fig 5:**
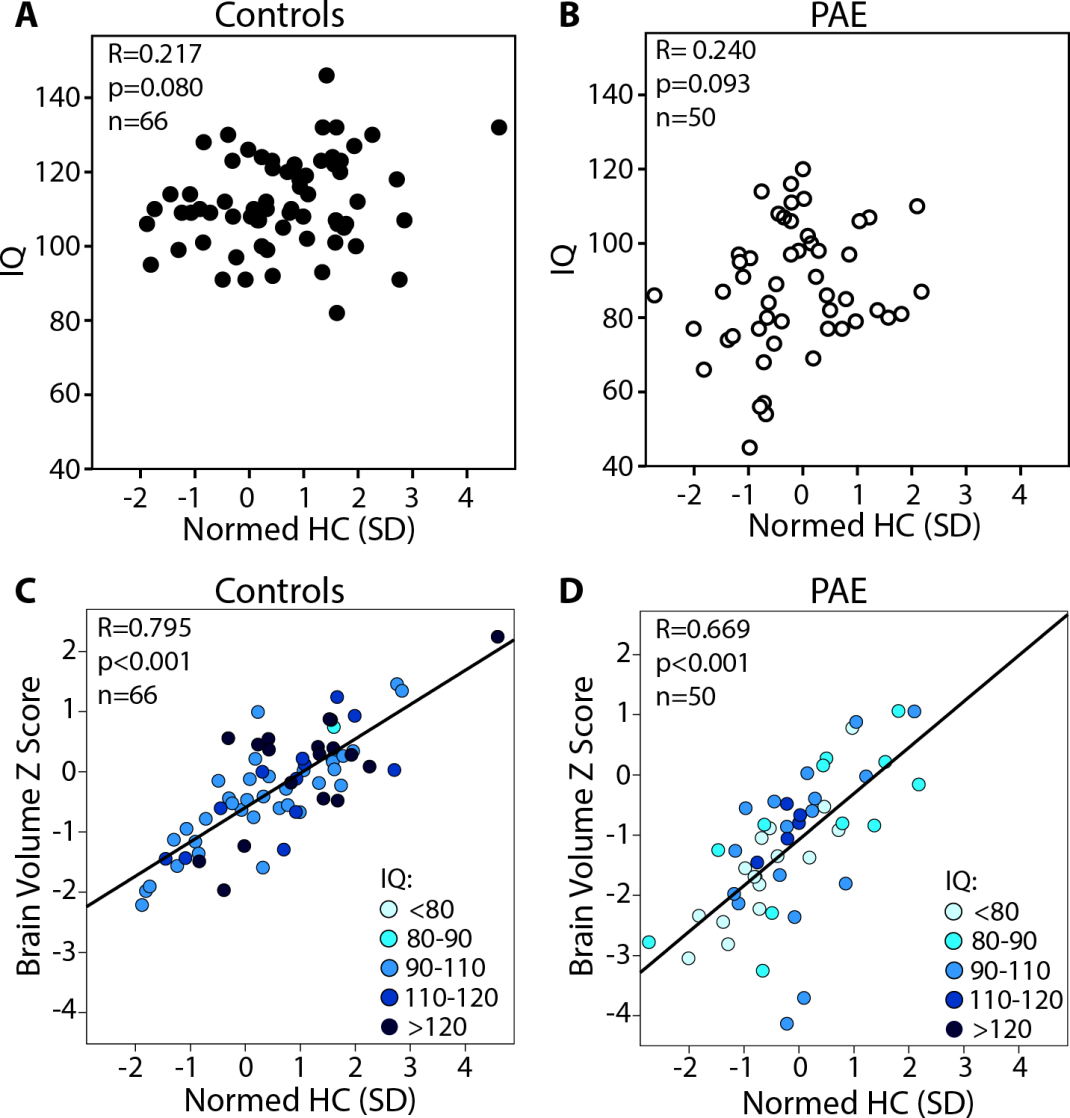
Age-standardized IQ versus normed head circumference standard deviation, showing non-significant relationships in both the control (A) and PAE groups (B), despite significant correlations between brain volume Z score and normed HC SD in this subset of participants (C,D). Likewise, there does not appear to be any systematic pattern/grouping of IQ in controls (C) or PAE (D) again demonstrating that those with the smallest normed HC and brain volume did not show consistently lower IQ scores.

Normed HC standard deviation and brain volume Z scores were positively correlated in both the control (R = 0.66, p<0.001) and PAE groups (R = 0.60, p<0.001), indicating that on the whole, subjects with larger HC for their age/sex have larger brain volumes (**Fig 4A and 4B**). However, this correlation did not hold among PAE participants with HC more than 1 SD below the norm (n = 34), or in all subjects (PAE and Control combined) with HC more than 1 SD below the norm (n = 49). Of note, similar R value correlations were found when analysis was repeated with partial correlations controlling for age (data not shown). IQ did not significantly correlate with normed HC (controls R = 0.22, p = 0.080; PAE R = 0.24 p = 0.093- **Fig 5A and 5B**), total brain volume Z scores (controls R = 0.09, p = 0.479; PAE R = 0.21, p = 0.146), or any lobe volume Z scores (controls R = -0.01–0.16, p = 0.957–0.202; PAE R = 0.19–0.26, p = 0.190–0.072). IQ was only available in 50 PAE and 66 control participants; however, this subsample had a similar age, sex and diagnostic sub-group distribution as the total sample (**Fig 2G and 2H**), and demonstrated tight correlations between brain volume Z scores and normed HC (**Fig 5C and 5D**), indicating that these negative findings (no relationships between HC or brain volume and IQ) are unlikely to stem from sample bias. Likewise, no other cognitive scores correlated with normed HC or total brain volume Z scores in either group, despite larger sample sizes of n~90–140 in each group.

### PAE Participants with Head Circumference Below the 3^rd^ Percentile

**Fig 6:**
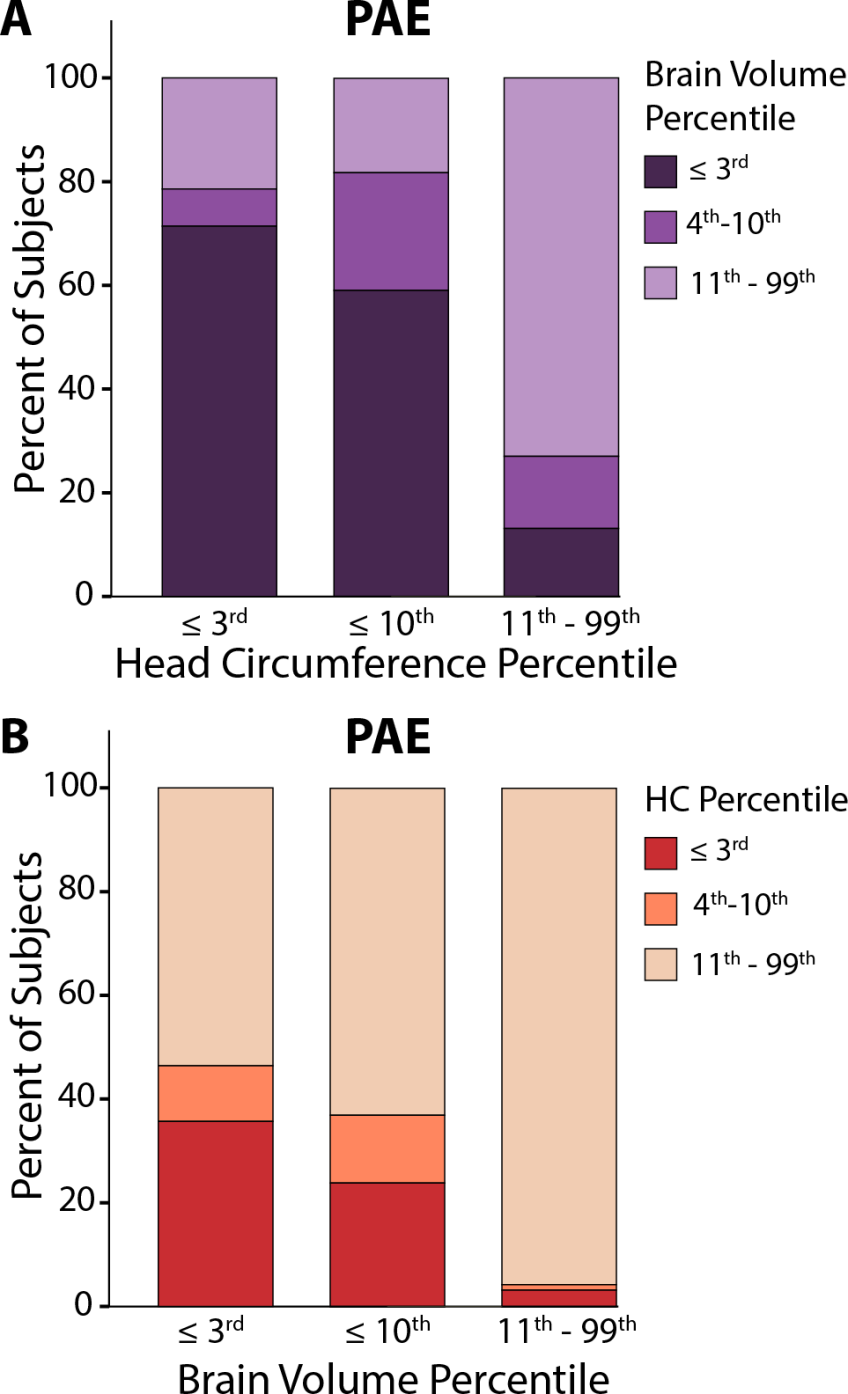
When only including the 14 PAE participants below the clinical cutoff for microcephaly (HC ≤ 3^rd^ percentile, A–column 1), it is notable that 10 (~70%) of the subjects have brain volumes below the 3^rd^ percentile. Similarly, ~80% of the subjects with HC ≤ 10^th^ percentile have brain volumes under the 10^th^ percentile (A–column 2). Conversely, among the PAE participants with total brain volume ≤ 3^rd^ (n = 14, B–column 1) or 10^th^ percentile (n = 22, B—column 2), 55–65% of subjects have HC in the ‘normal’ range from the 11^th^-99th percentiles, suggesting a disconnect between small brain volumes and head circumference. Note that the category of ≤ 10^th^ percentile on the x-axis of A and B includes subjects who are ≤ 3^rd^ percentile (to match cutoffs used in various diagnostic guidelines), while colour divisions within each bar are non-overlapping.

Only a small minority of PAE participants (14/144, 10%) met the clinical definition of microcephaly with HC ≤ 3^rd^ percentile. Of these 14 subjects, 11 (80%) had brain volume ≤3^rd^ percentile (**Fig 6A**). Similar proportions are observed for participants with HC ≤10^th^ percentile. Conversely, of the PAE participants with brain volume ≤3^rd^ percentile (n = 28), only ~35% had HC ≤3^rd^ percentile and more than half had HC percentiles in the normal range (**Fig 6B**). However, given that HC and brain volume were normed on different scales (population based sample versus control group, respectively) direct comparison may be confounded by group differences between our controls and the population norm. Nonetheless, only 50% subject overlap was found between the 14 subjects with HC ≤ 3^rd^ percentiles and the 14 subjects with the lowest brain volume percentiles in the PAE group, again suggesting a disconnect between these metrics. Cognitive test scores of the 14 PAE participants who had HC ≤3^rd^ percentile were not different than the whole PAE group (**Table 1**), suggesting that these participants are not more cognitively impaired than those PAE subjects with HC in the normal range.

## Discussion

This study provides evidence to support previous assertions of reduced head circumference in children and adolescents with prenatal alcohol exposure [14–18] by demonstrating: (i) a 29-point gap in median HC percentile, (ii) a 2-fold increase in the number of subjects with HC more than 1 SD below the population mean, and (iii) a nearly 5-fold increase in the number of subjects with HC ≤ 3^rd^ percentile in our PAE group compared to controls. This large sample of 144 PAE participants spans a wide range from 5 to 19 years of age, suggesting that HC deficits persist into young adulthood, in agreement with longitudinal studies of PAE [28]. Likewise, significant reductions of brain volume, IQ and cognitive performance are demonstrated in the PAE group, in keeping with previous literature in this population [29].

However, these results also highlight several important limitations of HC measurement in children with PAE. Despite group differences, the substantial spread in HC and its overlap between groups suggests that this metric does not discriminate individuals with PAE from healthy controls at a single-subject level. Moreover, although microcephaly (HC ≤3^rd^ percentile) occurred at a higher frequency in the PAE than control group, 90% of the PAE group had HC values above this clinical cutoff. Rates of microcephaly have previously been found to be low even in very large samples, e.g. 64 of 973 (~6.5%) PAE participants had HC<10^th^ percentile [12], again suggesting that microcephaly is not a sensitive marker of PAE.

In both the controls and PAE groups, HC is shown to positively correlate with brain volume; this relationship is not surprising—a child with a very low HC would be expected to have a smaller brain than a child on the high end of the HC range. However, increased variability in this relationship at the lower end of the HC range suggests that HC is a poor predictor of brain volume among groups of children with roughly similar normed HC values. Furthermore, lack of correlations between HC and cognitive measures suggests that HC does not predict functional impairment. As such, further investigation may be needed to determine if HC deficits are indeed a reflection of central nervous system impairment rather than overall growth deficiency.

Among the 14 PAE children with clinically significant microcephaly (HC≤ 3^rd^ percentile), there is no greater impairment in cognition (**Table 1**) and three participants have normal brain volumes (defined as above the 10^th^ percentile). Nonetheless, most (11/14) have brain volumes below 10^th^ percentile, as expected given that HC (i.e. the skull) poses a physical limitation on brain volume. However, it is important to note that the reverse relationship is not observed: about 60% of the subjects with very small brain volumes are shown to have normal HC values (again defined as above the 10^th^ percentile).

The lack of consistent correlation between brain volume and HC at low normed HC values may reflect greater variability in the ratio of brain tissue to cerebrospinal fluid in children with PAE, which could explain cases where brain volume is very small but HC is in the normal range. Conversely, reduced bone volume in the skull has been observed with micro computed tomography in a mouse model of FASD [30], and may account for cases where HC was reduced with intact brain volume. The skeletal and nervous systems are each uniquely affected by the toxic effects of alcohol in utero [31–33], likely adding variability to the relationship between brain volume and HC in children who were exposed to alcohol in varying amounts, frequencies and time-points throughout pregnancy. A recent study in autism found that non-neural tissue volume (skull, meninges, cerebrospinal fluid, etc.) was more highly correlated to HC than total brain volume, and that brain volume was only significantly correlated with HC in the control group (n = 26), but not the autism group (n = 34) [34], suggesting a disconnect in this relationship associated with another common neurodevelopmental disorder.

Beyond in utero sensitivity, the distinct post-natal trajectories of nervous and skeletal system development may also impact the relationship between HC and brain volume. HC increased with age at similar rates in both PAE and control groups, while brain volume did not change significantly in either group from 5–19 years, fitting with previous literature demonstrating that brain volume reaches ~90% of adult maximum at around age 6 years, while skull thickness continues to increase linearly with age into adolescence [35]. A recent study of healthy controls demonstrated that the brain-scalp distance increases with age in healthy children, driven primarily by increases in cerebrospinal fluid and cranial thickness [36], providing further evidence that HC reflects the composite of multiple systems that each develop at different rates with age. Nonetheless, when assessed separately, the developmental trajectories of HC and brain volume appear to be similar between the PAE and control groups, albeit in cross-sectional cohorts. Longitudinal samples may be better positioned to tease apart the relationship between these trajectories in PAE.

In addition to group differences, sex effects were observed for both brain volume and HC in the control group, as expected [37], but were less prominent between males and females in the PAE group (**Fig 2**). Both males and females with PAE showed significant brain volume reductions, albeit with a greater difference in males, in keeping with previous findings of more substantial brain volume reductions in males with FASD [24,38]. Head circumference was also reduced in males with PAE, but was not significantly lower in females with PAE, raising the question of the value of HC measurement particularly for females. Age-by-sex interactions have been observed in PAE studies of longitudinal cortical volume development [39], though the mechanisms underlying sex effects in PAE are unclear. Greater overlap in both HC and brain volume is observed between males and females in the PAE group across the whole age range, with no apparent divergence between sexes during adolescence. However, the effects of puberty were not tested but cannot be ruled out. Nonetheless, it remains possible that prenatal alcohol exposure has sex-specific impacts on early nervous system development, as observed in some animal models of PAE [40–42], and as suggested in developmental programming models of disease [43].

Several limitations of this study must be acknowledged. First, converting brain volumes to Z scores based on our control group (n = 145) may be less generalizable and more sensitive to sample bias than head circumference norms that were normalized here on a much larger published normative sample (n = 537) [1]. However, normative standards do not exist for brain volume, and thus the control sample was used here. Secondly, although weight was routinely collected prior to MRI acquisition, height was not consistently collected thus precluding examination of the effects of stature and/or growth deficiency on reduced head circumference in this population. Thirdly, our groups were imbalanced with respect to ethnicity, with 50% of our PAE group but only 3% of our control group self-identifying as aboriginal. Post-hoc analysis comparing brain volume Z scores and head circumference standard deviations between the aboriginal and non-aboriginal participants within the PAE group revealed no significant differences, suggesting that ethnicity does not have a strong effect on either metric. Moreover, group differences (PAE versus Control) and brain volume-head circumference correlations were re-tested after excluding aboriginal participants, which again yielded very similar results as those reported in our main study with ethnicity categories combined. Specifically, brain volume and head circumference were reduced (t = -7.4, p<0.001; t = -4.9, p<0.001, respectively) in non-aboriginal PAE participants (n = 72) compared to controls (n = 145). Likewise, brain volume Z score—head circumference SD correlations remained significant among non-aboriginal PAE participants (R = 0.611, p<0.001; n = 72), and again did not hold when including only those with head circumference below -1 SD from the population norm (R = 0.336, p = 0.187, n = 17). Lastly, although the lack of correlation between head circumference and brain volume among PAE participants with head circumference more than 1 SD below the population norm is intriguing, it is important to keep in mind that this reduced sample size (n = 34) limits power relative to the larger sample (n = 144) across the entire head circumference spectrum. Nonetheless, lack of correlation in this subset underscores the large inter-subject variability in this relationship, and provides further caution against its application to single-subject data for detecting central nervous system dysfunction (brain volumes or cognitive performance) based on head circumference.

Here we confirm previous reports of reduced head circumference, brain volume, and cognitive function in a large cohort of children with PAE relative to age and sex matched controls. Positive correlations are demonstrated between HC and brain volume, but the relationship weakens outside of the normal range in PAE, which may reflect the complex interplay between skeletal and neural development, each differentially affected by prenatal alcohol exposure. Further, although microcephaly is clearly more common in the PAE population, our findings suggest that it is only present in a small subset of children with PAE and does not co-occur with greater cognitive impairments.
